# Small islands and pandemic influenza: Potential benefits and limitations of travel volume reduction as a border control measure

**DOI:** 10.1186/1471-2334-9-160

**Published:** 2009-09-29

**Authors:** Martin Eichner, Markus Schwehm, Nick Wilson, Michael G Baker

**Affiliations:** 1 Department of Medical Biometry, University of Tübingen, Germany; 2 ExploSYS GmbH, Institute for Explorative Modeling, Leinfelden, Germany; 3 Department of Public Health, University of Otago, Wellington, New Zealand

## Abstract

**Background:**

Some island nations have explicit components of their influenza pandemic plans for providing travel warnings and restricting incoming travellers. But the potential value of such restrictions has not been quantified.

**Methods:**

We developed a probabilistic model and used parameters from a published model (i.e., *InfluSim*) and travel data from Pacific Island Countries and Territories (PICTs).

**Results:**

The results indicate that of the 17 PICTs with travel data, only six would be likely to escape a major pandemic with a viral strain of relatively low contagiousness (i.e., for *R*_0 _= 1.5) even when imposing very tight travel volume reductions of 99% throughout the course of the pandemic. For a more contagious viral strain (*R*_0 _= 2.25) only five PICTs would have a probability of over 50% to escape. The total number of travellers during the pandemic must not exceed 115 (for *R*_0 _= 3.0) or 380 (for *R*_0 _= 1.5) if a PICT aims to keep the probability of pandemic arrival below 50%.

**Conclusion:**

These results suggest that relatively few island nations could successfully rely on intensive travel volume restrictions alone to avoid the arrival of pandemic influenza (or subsequent waves). Therefore most island nations may need to plan for multiple additional interventions (e.g., screening and quarantine) to raise the probability of remaining pandemic free or achieving substantial delay in pandemic arrival.

## Background

There were large (voluntary) reductions of travel volumes associated with the global spread of severe acute respiratory syndrome (SARS) [1]. There have also been media reports of reduced tourist flows associated with the swine-origin (H1N1) influenza pandemic during 2009 (particularly for Mexico). Such a phenomenon may reoccur with the emergence of more virulent waves of the current pandemic or if new strains of pandemic influenza emerge.

In addition to voluntary changes in travel volumes, governments may also impose legal restrictions on travel and use exit and entry screening. Indeed, some island nations have explicit components of their influenza pandemic plans for providing travel warnings and restricting incoming travellers e.g., New Zealand [2] and all four Pacific Island Countries and Territories (PICTs) with published plans that were examined in a recent review [3]. Furthermore, some modelling work suggests that international air travel restrictions may contribute to delaying the global spread of a pandemic [4].

While a World Health Organization Writing Group [5] recognised that islands have achieved border control successes with pandemic influenza in the past, a more recent review cited expert opinion against the use of mandatory travel restrictions for pandemic influenza control [6]. However this review appeared to be in the context of large countries and did not consider islands (especially low-income island nations which cannot necessarily afford some other control options). Therefore, to better guide the use of these interventions, we aimed to quantify the potential impact of travel volume reductions to prevent (or at least delay) the entry of pandemic influenza into small Pacific island nations.

## Methods

### Model of a global pandemic and assumptions

We considered that a global influenza pandemic would spread around the world via aircraft travel and have an average reproduction number (*R*_0_) in the range of 1.5 to 3.0 (with a mid-range value of 2.25). The pandemic was assumed to be in the form of a single pandemic wave that would end within a year.

For this pandemic scenario, we developed a probabilistic mathematical model that is described in detail in the Technical Appendix (Additional file 1) along with a numerical example for one island nation. An interactive software application that was based on this model was also developed and is freely available online http://www.influsim.info/software/escaval[7].

The key parameter calculated was the "island escape probability" which was the probability that an island nation would avoid an outbreak of pandemic influenza for the full course of the global pandemic. The values of the input parameters used in our model for the global pandemic were based on the published model *InfluSim *[8] (with version 2.1, April 2008, being freely downloadable). In addition, we made the two other assumptions to increase model realism:

• Only 50% of "moderately sick" cases (as defined in [8]) were assumed to be well enough to travel.

• Only 10% of "severely sick" cases (as defined in [8]) were assumed to be well enough to travel.

Such assumptions are likely to be very conservative as they assume no exit screening by pandemic-affected nations. We also assumed that no other pandemic influenza control measures would be utilized in the island nations. That is, no entry screening; no provision of antivirals to travellers; no quarantine; and no use of pre-pandemic vaccine or of a vaccine that had been developed after the emergence of the new pandemic strain.

### Assumptions on travel reductions

We assumed that voluntary travel reductions (averaged over the course of the pandemic) might be similar to those experienced during SARS for travel between Hong Kong and the United States at 79% [9]. Much higher levels of travel volume reduction (i.e., 99%) were assumed to relate to restrictions imposed by governments of island nations and to reflect essential diplomatic and emergency travel only (or complete "official" border closure with some leakage attributable to illegal yacht movements and private plane use).

### Travel data

We collected travel volume data for all the PICTs that were: (i) members of the Secretariat of the Pacific Community (SPC); (ii) which had a population of under one million (which excluded Papua New Guinea); and (iii) which had an airport (i.e., which excluded Tokelau and Pitcairn Island). Data were from the SPC website [10] and from its links to the websites of the Statistics Departments/Ministries of the PICTs (where these existed).

### Calculating the escape probability

Using these data, we expect that a given number of infected individuals enter the island during the global pandemic. Depending on their course of disease and on the remaining time of contagiousness, the expected number of secondary cases per index case varies, and so does the probability that the index case triggers a major outbreak on the island. We combine all possible events, taking into consideration their individual probabilities, to calculate the probability that an island will either experience a major outbreak or ultimately escape the pandemic. Our calculations assume that travel restrictions are performed from the very beginning of a pandemic until the end or until the failure to prevent introduction becomes evident.

## Results

The results (Table 1) indicate that for the 17 PICTs with travel data, only six would be likely to avoid introduction of pandemic influenza, even if the pandemic strain was of relatively low contagiousness (i.e., for *R*_0 _= 1.5) and if very tight travel reductions of 99% were applied throughout the course of the global pandemic (Table 1). For more severe pandemics (*R*_0 _= 2.25 or higher), only four to five PICTs would have more than 50% probability of escaping. Only one country (Tuvalu) was considered to have a high chance of escaping a relatively "mild" pandemic by relying on voluntary travel volume reductions alone (i.e., a 79% reduction level).

**Table 1:** Probability of small islands in the South Pacific escaping a global influenza pandemic (for different values of *R*_0 _and different travel volume reductions for arriving travellers).

Country (year for traveler arrival data)	Total annual traveler arrivals	Island escape probability for global influenza pandemic					
		99% travel reduction			79% travel reduction		
		*R*_0 _= 1.5	*R*_0 _= 2.25	*R*_0 _= 3.0	*R*_0 _= 1.5	*R*_0 _= 2.25	*R*_0 _= 3.0
Guam (2007/08)	1,210,600†	<0.01	<0.01	<0.01	<0.01	<0.01	<0.01
Fiji (2004)	596,084	<0.01	<0.01	<0.01	<0.01	<0.01	<0.01
Northern Mariana Islands (2004)	589,244*	<0.01	<0.01	<0.01	<0.01	<0.01	<0.01
French Polynesia (2006)	221,549*	0.02	<0.01	<0.01	<0.01	<0.01	<0.01
Samoa (2007)	196,627‡	0.03	<0.01	<0.01	<0.01	<0.01	<0.01
Vanuatu (2006)	154,101§	0.06	<0.01	<0.01	<0.01	<0.01	<0.01
Cook Islands (2007)	109,115	*0.14*	<0.01	<0.01	<0.01	<0.01	<0.01
New Caledonia (2006)	100,491*	*0.16*	0.01	<0.01	<0.01	<0.01	<0.01
Palau (2006)	86,375*	*0.21*	0.02	<0.01	<0.01	<0.01	<0.01
American Samoa (2006)	72,800	*0.27*	0.04	0.01	<0.01	<0.01	<0.01
Tonga (2003)	63,451¶	*0.32*	0.06	0.02	<0.01	<0.01	<0.01
Federated States of Micronesia (FSM) (2005)	18,958*	**0.71**	*0.43*	*0.32*	<0.01	<0.01	<0.01
Solomon Islands (2007)	13,748*	**0.78**	**0.54**	*0.44*	<0.01	<0.01	<0.01
Marshall Islands (2005)	9173*	**0.85**	**0.66**	**0.57**	0.03	<0.01	<0.01
Kiribati (2006)	4704#	**0.92**	**0.81**	**0.75**	*0.17*	0.01	<0.01
Niue (2006)	4588**	**0.92**	**0.81**	**0.76**	*0.18*	0.01	<0.01
Tuvalu (2007)	1130	**0.98**	**0.95**	**0.93**	**0.65**	*0.34*	*0.24*
Nauru	n/a	-	-	-	-	-	-
Wallis and Futuna	n/a	-	-	-	-	-	-

Notes:

The coding of the island escape probability: standard type, <10%; italics, 10 - 50%; bold, > 50%.

* Available data do not include island citizens returning from overseas travel (i.e., non-citizen arrivals only) though the former are usually a small proportion of arrivals relative to non-citizens for most PICTs.

† For Guam this figure was extrapolated from arrivals data for October 2007 to February 2008. Includes civilian and armed forces arrivals by air (not by sea) and does not include data on returning citizens of Guam.

‡ For Samoa this figure involves an extrapolation of arrivals data for January to July 2007 and includes a figure for returning residents based on the assumption that all Samoan citizens who departed returned in the same year (2005 data). These figures include arrivals by sea (4.4% of total visitor arrivals).

§For Vanuatu this figure includes day visitors from ships (n = 85,922).

¶For Tonga this figure includes an estimate for returning residents and arrivals by ship and yacht.

# For Kiribati this figure includes an estimate for returning residents based on the assumption that all "I-Kiribati" leaving also return in the same year (n = 130 for the most recent year with data i.e., 2002).

** For Niue this figure includes an estimate for returning residents based on the assumption that all "residents not departing permanently" return in the same year.

n/a: No data available.

Two of the 19 PICTs had no travel data and for the others much of the data were suboptimal in that they did not always include numbers of returning citizens, and often only the arrivals by air (i.e., ignoring arrivals by sea; for details, see Table 1).

**Figure 1:**
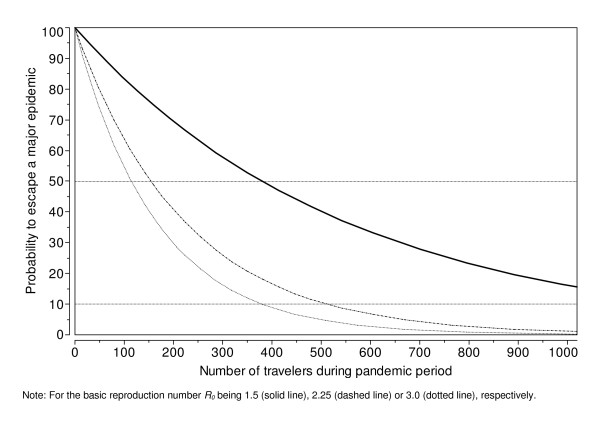
**Probability to escape a major epidemic by number of visitors arriving on a PICT during the pandemic period**.

Figure 1 shows how the island escape probability depends on the total number of travellers arriving on a PICT during the course of the global pandemic. For *R*_0 _= 1.5, the critical number of travellers must not exceed 380, if the PICT aims to have an escape probability above 50%. For *R*_0 _= 2.25 and *R*_0 _= 3.0, these critical values are 155 and 115 travellers respectively.

Severely or moderately sick travellers were assumed to have a reduced probability of travel. Because of this, and because of the large fraction of individuals who remain asymptomatic througout the course of their infection, nearly 75% of infected visitors do not show any symptoms upon arrival on a PICT. This value only depends on the natural history of the disease and on the propensity of sick people to travel, but it is independent on *R*_0 _(see the Technical Appendix for more details).

## Discussion

### Main findings and interpretation

This analysis suggests that only a few PICTs might be expected to avoid pandemic influenza by relying on extremely rigorous travel volume reductions alone. Consequently, most PICTs need to consider multiple additional options in their pandemic planning (especially for pandemics with high case fatality ratios). These measures might include: entry screening using health questionnaires and use of rapid diagnostic tests; routine facility quarantine [11] or home quarantine with intensive monitoring; possibly the routine provision of antivirals to incoming travellers; pre-pandemic vaccination of their populations (if an appropriate vaccine became available); enhanced capacity for disease surveillance in the community and for rapid outbreak control capacity. As nearly 75% of infected travellers arrive without symptoms, entry screening based on the travellers' symptom states alone only slightly improves the escape probability (e.g. it increases Tonga's escape probability from 32 to 46% for the *R*_0 _= 1.5 scenario with 99% travel reduction) if all symptomatic travellers are prevented from infecting anybody.

Our calculations assume that travel reduction remains constant during the whole period of the global pandemic. Higher numbers of travellers may temporarily be admitted from regions which are not or only slightly afflicted by the pandemic, but this strategy may be too difficult to implement because it would require the travel history of each arriving traveler to be verified. An alternative to these interventions is planning for complete border closure (i.e., practically 100% travel volume reduction) at the first sign of a global pandemic - a response that some PICTs used successfully during the 1918/19 influenza pandemic [12].

Even rigorous travel volume reductions might, however, be difficult for those PICTs that partially depend on food imports and other critical imports (e.g., medical supplies). Nevertheless, some PICTs might be able to facilitate ongoing trade by aircraft and shipping while keeping the crews of these vessels entirely separated from the local population (e.g., with high security unloading facilities where the crew never actually disembark while their vessel is unloaded). Others could enhance food self-sufficiency by increasing fishing and diverting export crops (e.g., coconut oil) for use as food.

Pandemic severity varies greatly with the experience of the current swine-origin (H1N1) influenza pandemic (at least to mid-2009) indicating a severity that might even be less overall than seasonal influenza. Therefore, good data on severity at the start of a pandemic (or a new pandemic wave) will help island nations decide if mandated travel volume reduction is a worthwhile intervention. Key variables for such early decisions from affected countries (especially developing countries) are hospitalisation rates and case fatality ratios.

Also of note that some actions that would assist with severe travel volume reductions during pandemic influenza might be worthwhile in their own right. One example is building infrastructure to improve access to the Internet and to allow videoconferencing. Diversifying island economies (to reduce reliance on tourism) may also cushion island economies against other natural disasters and routine fluctuations in tourism numbers.

Although travel restrictions may not be sufficient to prevent the successful importation of an infection, they should (at least on average) delay it. Scalia Tomba and Wallinga [13] demonstrated with a simple mathematical model that an overall travel reduction by 99% should delay an epidemic on an island by about three weeks if R_0 _is approximately 2.

### Limitations

This analysis made many simplifying assumptions. It could potentially be improved by developing a more complex stochastic model that used log-normal or gamma-distributed sojourn times (rather than the exponential distributions used here). Such a model would also be able to provide information on the average time of pandemic arrival. Improved modelling (including combining additional border control interventions with travel reductions) may not only facilitate pandemic planning among PICTs but also help other island nations (and sub-national island jurisdictions) in the Caribbean, Southeast Asia and off the coast of most continents.

Although we considered a range of values of *R*_0_, it is conceivable that in a global pandemic the effective *R*_0 _would decline after the first few months of pandemic emergence. This decline is because many countries around the world are very likely to adopt social distancing and other control measures. Possibly after some months, relevant technologies such as a pandemic strain vaccine might also become available (and start to be used by those planning to travel). Indeed, arriving travellers might be required to show a certificate of vaccination with a new pandemic vaccine.

The missing and suboptimal nature of some of the travel data shown in Table 1 is problematic. There is a need for regional agencies to encourage improved data collection and publication by PICTs so that studies such as the one reported here can be undertaken with more realism.

Finally we note that this work has not assessed the value to policy makers of island nations in presenting "escape probabilities" in the way that we have. It could be that to use a mandated travel reduction intervention, policy makers would need to have indications of much higher rates of success than thresholds of 50% (as used in Table 1). They may also need indications of the potential number of hospitalisations prevented and lives saved before there is political and popular acceptance of the policy (e.g. as calculated from case fatality ratios in other countries).

## Conclusion

These results suggest that relatively few island nations could successfully rely on intensive travel volume restrictions alone to avoid the arrival of pandemic influenza. Therefore most island nations will need to plan for multiple additional interventions (e.g., screening and quarantine) to raise the probability of remaining pandemic free.

## Competing interests

The authors declare that they have no competing interests.

## Authors' contributions

This study was conceived by NW and MB. ME developed the modelling approach and the mathematics. MS designed the software. NW collected the input data. All authors contributed to drafts of the manuscript.

## Pre-publication history

The pre-publication history for this paper can be accessed here:

http://www.biomedcentral.com/1471-2334/9/160/prepub

## Supplementary Material

Additional file 1 **"Technical Appendix: Formulae for considering pandemic influenza importation into islands"**. The technical appendix provides the detailed formulae data for the model used for assessing pandemic influenza importation into islands. Click here for file
