# Supplementary material for: Small islands and pandemic influenza: Potential benefits and limitations of travel volume reduction as a border control measure
Source: BMC Infect Dis. 2009 Sep 29;9:160. doi: 10.1186/1471-2334-9-160 (PMC2761921; doi:10.1186/1471-2334-9-160)
Supplement: Additional file 1 — "Technical Appendix: Formulae for considering pandemic influenza importation into islands". The technical appendix provides the detailed formulae data for the model used for assessing pandemic influenza importation into islands. [file 1471-2334-9-160-S1.DOC]

**Technical Appendix: Formulae for considering pandemic influenza importation into islands**

## Global infection attack rate

The fraction $f_{\infty}$ of individuals who finally experience infection is given by the implicit formula:$R_{0}f_{\infty}=-\ln(1-f_{\infty})$. This formula assumes that the whole population is initially susceptible and that the basic reproduction number $R_{0}$ is constant during the full time period of the global pandemic.

## Natural history of disease

Each infected individual is assumed to pass through three different stages:

1. latent period: exponentially distributed with average duration $D_{L}$
2. prodromal period: exponentially distributed with average duration $D_{P}$
3. fully infectious period: exponentially distributed with average duration $D_{I}$
   individuals in this period can experience three different types of symptoms
   1. a fraction $f_{A}$ experiences asymptomatic infection,
   2. a fraction $f_{M}$ experiences moderate disease,
   3. a fraction $f_{S}$ experiences severe disease.

## Contagiousness of infected individuals

We assume that:

1. the contagiousness of infected individuals is zero in the latent period,
2. their relative contagiousness is $\tilde{c}_{P}$ in the prodromal fever period,
3. the relative contagiousness of asymptomatic individuals is $\tilde{c}_{A}$ ,
4. the relative contagiousness of moderately sick cases is $\tilde{c}_{M}$ ,
5. the relative contagiousness of severely sick cases is $\tilde{c}_{S}$.

The contagiousness of infected individuals now needs to be normalized in order to obtain the chosen effective reproduction number $R_{0}$.

Using , we calculate

1. the absolute contagiousness during the prodromal period as $c_{P}=x \tilde{c}_{P}$,
2. the absolute contagiousness of asymptomatic individuals as $c_{A}=x \tilde{c}_{A}$,
3. the absolute contagiousness of moderately sick cases as $c_{M}=x \tilde{c}_{M}$,
4. the absolute contagiousness of severely sick cases as $c_{S}=x \tilde{c}_{S}$,

and we obtain $c_{P}D_{P}+c_{A}f_{A}D_{I}+c_{M}f_{M}D_{I}+c_{S}f_{S}D_{I}=R_{0}$.

## Probability of randomly picking an infected individual for traveling

Given a time window of length $W$which fully contains the global pandemic, the probability that a randomly chosen individual is infected is$f_{\infty}(D_{L}+D_{P}+D_{I})/W$if all individuals are equally likely to travel. This probability can be broken down in the probability of:

1. picking an individual during the incubation period: $i_{L}=f_{\infty}D_{L}/W$,
2. picking an individual during the prodromal period: $i_{P}=f_{\infty}D_{P}/W$,
3. picking an asymptomatically infected individual: $i_{A}=f_{\infty}D_{I}f_{A}/ W$.

If a fraction $T_{M}$ or $T_{S}$moderately and severely sick travelers, respectively, decide not to travel, we get the probability of:

1. picking a moderately infected individual: $i_{M}=f_{\infty}D_{I}f_{M}(1-T_{M})/ W$,
2. picking a severely infected individual: $i_{S}=f_{\infty}D_{I}f_{S}(1-T_{S})/ W$.

## Fraction of travelers entering the country in a given infection stage

Symptom screening for all arriving passengers can be implemented by rejecting a fraction $B_{M}$ (or $B_{S}$) of moderately (or severely) sick passengers upon arrival (or by effectively placing such travelers into fully effective isolation facilities). Combining each traveler’s probability to be infected ($i_{L},...,i_{S}$) with disease progression during traveling and with the rejection of symptomatic travelers at the border ($B_{M}$ and $B_{S}$), we obtain the probability that infected individuals effectively enter the island and mingle with the population as follows:

1. $t_{L}=i_{L}q_{L\to L}$ travelers arrive in their latent period
2. $t_{P}=i_{L}q_{L\to P}+i_{P}q_{P\to P}$ travelers arrive in their prodromal period
3. $t_{A}=i_{L}q_{L\to A}+i_{P}q_{P\to A}+i_{A}q_{I\to I}$ asymptomatic travelers arrive
4. $t_{M}=(i_{L}q_{L\to M}+i_{P}q_{P\to M}+i_{M}q_{I\to I})(1-B_{M})$ moderately sick travelers arrive
5. $t_{S}=(i_{L}q_{L\to S}+i_{P}q_{P\to S}+i_{S}q_{I\to I})(1-B_{S})$ severely sick travelers arrive

The disease progression quantities $q_{X\to Y}$ can be derived from the system of differential equations $dL/dt=-L/D_{L}$, $dP/dt=L/D_{L}-P/D_{P}$ and $dI/dt=f_{I}P/D_{P}-I/D_{I}$ where *L* and *P* are the number of infected individuals in the latent and prodromal stage, respectively, and where *I* can stand for asymptomatic (index *A*), moderately sick (index *M*) or severely sick (index *S*) individuals. They depend on the duration of traveling $D_{V}$ as follows:

$q_{L\to L}=e^{-D_{V}/D_{L}}$, $q_{P\to P}=e^{-D_{V}/D_{P}}$, $q_{I\to I}=e^{-D_{V}/D_{I}}$,

$q_{L\to P}=\frac{1/D_{L}}{1/D_{L}-1/D_{P}}\left(e^{-D_{V}/D_{P}}-e^{-D_{V}/D_{L}}\right)$, $q_{P\to I}=f_{I}\frac{1/D_{P}}{1/D_{P}-1/D_{I}}\left(e^{-D_{V}/D_{I}}-e^{-D_{V}/D_{P}}\right)$,

$q_{L\to I}=f_{I}\frac{1/(D_{_{L}}D_{P})}{1/D_{L}-1/D_{P}}\left(\frac{e^{-D_{V}/D_{I}}-e^{-D_{V}/D_{P}}}{1/D_{P}-1/D_{I}}-\frac{e^{-D_{V}/D_{I}}-e^{-D_{V}/D_{L}}}{1/D_{L}-1/D_{I}}\right)$.

## Probability that a given number of infected travelers enter an island

We now assume that a fraction $T_{all}$ of travelers will be prevented during the pandemic wave from entering the island. Using the binomial distribution, we derive

1. the probability that *k* individuals arrive with infection in the latent stage:
$p_{L}^{A}(i)=\left(\begin{matrix} N(1-T_{all}) \\ k \end{matrix}\right)t_{L}^{k}\left(1-t_{L}\right)^{N(1-T_{all})-k}$
2. the probability that *k* individuals arrive with infection in the prodromal stage:
$p_{P}^{A}(i)=\left(\begin{matrix} N(1-T_{all}) \\ k \end{matrix}\right)t_{P}^{k}\left(1-t_{P}\right)^{N(1-T_{all})-k}$
3. the probability that *k* individuals arrive in the asymptomatic stage:
$p_{A}^{A}(i)=\left(\begin{matrix} N(1-T_{all}) \\ k \end{matrix}\right)t_{A}^{k}\left(1-t_{A}\right)^{N(1-T_{all})-k}$
4. the probability that *k* individuals arrive moderately sick:
$p_{M}^{A}(i)=\left(\begin{matrix} N(1-T_{all}) \\ k \end{matrix}\right)t_{M}^{k}\left(1-t_{M}\right)^{N(1-T_{all})-k}$
5. the probability that *k* individuals arrive severely sick:
$p_{S}^{A}(i)=\left(\begin{matrix} N(1-T_{all}) \\ k \end{matrix}\right)t_{S}^{k}\left(1-t_{S}\right)^{N(1-T_{all})-k}$

## Symptomatic stage of arriving infected travelers

We expect that a fraction $\frac{t_{L}+t_{P}+t_{A}}{t_{L}+t_{P}+t_{A}+t_{M}+t_{S}}$of all infected travelers who enter the island do not show any symptoms. This result does not depend on the basic reproduction number $R_{0}$, nor on the size of the time window $W$, nor on the number of travelers per year $N$, nor on the general travel reduction $T_{all}$.

## Expected number of secondary infections caused by travelers after arrival

Depending on the state of infection and disease, newly arrived travelers have a different value for “remaining contagiousness”.

1. Individuals who arrive in their latent stage still have their full period of contagiousness ahead of them and cause on average $R_{0}$ secondary infections after their arrival.
2. As a result of assuming an exponentially distributed sojourn time, individuals who arrive in their prodromal period, still have their full period of contagiousness ahead of them and also cause an average of $R_{0}$ secondary infections after their arrival.
3. Individuals who arrive in their fully infectious period, have already passed through their prodromal period and therefore, cause on average
   1. $R_{A}=c_{A}D_{A}$ infections if they are infected asymptomatically,
   2. $R_{M}=c_{M}D_{M}$ infections if they are moderately sick,
   3. $R_{S}=c_{S}D_{S}$ infections if they are severely sick.

## Probability that a major epidemic is triggered by travelers

The probability that the island does not develop a major epidemic when exposed to a single infected person in the latent or prodromal stage is 1/$R_{0}$. The probability to escape an epidemic in spite of *k* such people spreading the infection is $R_{0}^{-k}$. Therefore, the probability that the island *escapes* a major epidemic

1. despite all the individuals entering in their latent period is $p_{L}^{E}=\sum_{k=0}^{\infty} \left(\begin{matrix} N(1-T_{all}) \\ k \end{matrix}\right)t_{L}^{k}\left(1-t_{L}\right)^{N(1-T_{all})-k}R_{0}^{-k}=\left(1+t_{L}\left(\frac{1}{R_{0}}-1\right)\right)^{N(1-T_{all})}$
2. despite all the individuals entering in their prodromal period is $p_{P}^{E}=\left(1+t_{P}\left(\frac{1}{R_{0}}-1\right)\right)^{N(1-T_{all})}$.

Individuals entering the island after the prodromal period are no longer expected to cause the full number of $R_{0}$secondary cases, but $R_{A}$, $R_{M}$ or $R_{S}$ secondary infections, respectively, depending on their course of disease. The number of secondary cases of each one of these individuals follows a geometric distribution, e. g. the probability that an asymptomatically infected individual infects *i* others is $R_{A}^{i}/(1+R_{A})^{i+1}$. As each one of their secondary cases is expected to infect $R_{0}$ others, we can calculate the probability that the island experiences a major epidemic by using the secondary cases instead of using the travelers with diminished infection potential. The probability that the island *escapes* a major epidemic

1. despite individuals entering asymptomatically infected is $p_{A}^{E}=\sum_{k=0}^{\infty} \left(\begin{matrix} N(1-T_{all}) \\ k \end{matrix}\right)t_{A}^{k}\left(1-t_{A}\right)^{N(1-T_{all})-k}\left(\sum_{i=0}^{\infty} \frac{R_{A}^{i}}{(1+R_{A})^{i+1}}R_{0}^{-i}\right)^{k}=\left(1+t_{A}\left(\frac{1}{1+R_{A}(1-1/R_{0})}-1\right)\right)^{N(1-T_{all})}$
2. despite individuals entering moderately sick is $p_{M}^{E}=\left(1+t_{M}\left(\frac{1}{1+R_{M}(1-1/R_{0})}-1\right)\right)^{N(1-T_{all})}$
3. despite individuals entering severely sick is $p_{S}^{E}=\left(1+t_{S}\left(\frac{1}{1+R_{S}(1-1/R_{0})}-1\right)\right)^{N(1-T_{all})}$

Combining all escape probabilities gives the probability that the island will escape a major epidemic caused by any traveler: $p^{E}=p_{L}^{E}\cdot p_{P}^{E}\cdot p_{A}^{E}\cdot p_{M}^{E}\cdot p_{S}^{E}$.

**Numerical example: American Samoa**

To give a numerical example of the calculations, we have chosen American Samoa which normally receives 72,800 travelers a year (see Table 1 in the main text). We assume that the number of travelers will either drop by 79% (voluntary reduction) or by 99% (government enforced reduction) during the period of the pandemic (which lasts one year). The results of the calculations are summarized in Table A2. Although the average fractions of infected travelers are very low, more than 100 infected individuals enter the country if travel is only reduced by 79%. If incoming travel is reduced by 99% instead (i.e. if only 728 travelers are allowed to enter the island), the expected number of people who arrive infected drops to 6.6 which is still too large. The probability that American Samoa will be spared a major epidemic under these assumptions is still only 3.8% (the probability to escape a major outbreak is given by the product of the individual escape probabilities: 0.396*0.658*0.395*0.481*0.767 = 0.038 for 99% travel reduction). With 96.2% probability the infection will break through in spite of the control measures. A fraction of 73.2% of all infected visitors do not show any symptoms upon arrival, rendering any border control measures based on symptoms alone futile. If symptomatic cases are rejected or isolated upon entry, the escape probability has to be calculated with the first three terms only (e.g. 0.396*0.658*0.395 = 0.103 for 99% travel reduction and full border control).

|  | **Latent  period** | **Prodromal period** | **Asymp-tomatic infection** | **Moderately**  **sick** | **Severely sick** |
| --- | --- | --- | --- | --- | --- |
| Average duration [days] | $D_{L}=1.4$ | $D_{P}=0.5$ | $D_{I}=4$ | $D_{I}=4$ | $D_{I}=4$ |
| Fraction of infected | all | all | $f_{A}=1/3$ | $f_{M}=1/3$ | $f_{S}=1/3$ |
| Fraction too sick to travel | none | none | none | $T_{M}=0.5$ | $T_{S}=0.9$ |
| Relative contagiousness | none | $\tilde{c}_{P}=0.5$ | $\tilde{c}_{A}=0.5$ | $\tilde{c}_{M}=1.0$ | $\tilde{c}_{S}=1.0$ |
| Rejected at the border | none | none | none | $B_{M}=0$ | $B_{S}=0$ |

Table A1. Parameter values.
It is assumed that traveling to a particular island nation takes 12 hours; basic reproduction number *R_0_* is varied from 1.5 to 3.0.

|  | **Latent  period** | **Prodromal period** | **Asymp-tomatic infection** | **Moderate­ly sick** | **Severely sick** |
| --- | --- | --- | --- | --- | --- |
| Probability that a random­ly choosing traveler is in the given state | $\begin{matrix} i_{L} \\ 0.33\% \end{matrix}$ | $\begin{matrix} i_{P} \\ 0.12\% \end{matrix}$ | $\begin{matrix} i_{A} \\ 0.31\% \end{matrix}$ | $\begin{matrix} i_{M} \\ 0.16\% \end{matrix}$ | $\begin{matrix} i_{S} \\ 0.03\% \end{matrix}$ |
| Probability that a tra­ve­ler is in the given state when entering American Samoa | $\begin{matrix} t_{L} \\ 0.23\% \end{matrix}$ | $\begin{matrix} t_{P} \\ 0.10\% \end{matrix}$ | $\begin{matrix} t_{A} \\ 0.31\% \end{matrix}$ | $\begin{matrix} t_{M} \\ 0.17\% \end{matrix}$ | $\begin{matrix} t_{S} \\ 0.06\% \end{matrix}$ |
| Expected number of tra­ve­lers in the given state during the pandemic; 79% reduction | $\begin{matrix} N_{L}^{79\%} \\ 35.0 \end{matrix}$ | $\begin{matrix} N_{P}^{79\%} \\ 15.8 \end{matrix}$ | $\begin{matrix} N_{A}^{79\%} \\ 47.4 \end{matrix}$ | $\begin{matrix} N_{M}^{79\%} \\ 26.4 \end{matrix}$ | $\begin{matrix} N_{S}^{79\%} \\ 9.6 \end{matrix}$ |
| Probability that none of these travelers starts an epidemic | $\begin{matrix} p_{L}^{79\%} \\ <0.1\% \end{matrix}$ | $\begin{matrix} p_{P}^{79\%} \\ <0.1\% \end{matrix}$ | $\begin{matrix} p_{A}^{79\%} \\ <0.1\% \end{matrix}$ | $\begin{matrix} p_{M}^{79\%} \\ <0.1\% \end{matrix}$ | $\begin{matrix} p_{S}^{79\%} \\ 0.38\% \end{matrix}$ |
| Expected number of tra­ve­lers in the given state during the pandemic; 99% reduction | $\begin{matrix} N_{L}^{99\%} \\ 1.7 \end{matrix}$ | $\begin{matrix} N_{P}^{99\%} \\ 0.8 \end{matrix}$ | $\begin{matrix} N_{A}^{99\%} \\ 2.3 \end{matrix}$ | $\begin{matrix} N_{M}^{99\%} \\ 1.3 \end{matrix}$ | $\begin{matrix} N_{S}^{99\%} \\ 0.5 \end{matrix}$ |
| Probability that none of these travelers starts an epidemic | $\begin{matrix} p_{L}^{99\%} \\ 39.6\% \end{matrix}$ | $\begin{matrix} p_{P}^{99\%} \\ 65.8\% \end{matrix}$ | $\begin{matrix} p_{A}^{99\%} \\ 39.5\% \end{matrix}$ | $\begin{matrix} p_{M}^{99\%} \\ 48.1\% \end{matrix}$ | $\begin{matrix} p_{S}^{99\%} \\ 76.7\% \end{matrix}$ |

Table A2. Expected results for American Samoa.

The calculations are based on an effective reproduction number $R_{0}=2.25$, which corresponds to an infection attack rate of $f_{\infty}=85.3\%$. During one year, 72,800 travelers usually arrive in American Samoa, but travel is reduced by either 79% (voluntary reduction) or by 99% (government enforced reduction) during the pandemic which is assumed to last for $W=1$ year (for other parameter values, see Table A1).
